# Contextual factors and clinical reasoning: differences in diagnostic and therapeutic reasoning in board certified versus resident physicians

**DOI:** 10.1186/s12909-017-1041-x

**Published:** 2017-11-15

**Authors:** Elexis McBee, Temple Ratcliffe, Katherine Picho, Lambert Schuwirth, Anthony R. Artino, Ana Monica Yepes-Rios, Jennifer Masel, Cees van der Vleuten, Steven J. Durning

**Affiliations:** 1 Department of Medicine, Naval Medical Center San Diego, 34800 Bob Wilson Drive, San Diego, 92134 California USA; 2 Department of Medicine, University of Texas Health Science Center at San Antonio, 7703 Floyd Curl Drive, San Antonio, 78229 Texas USA; 3 Department of Medicine, F. Edward Hébert School Of Medicine, Uniformed Services University, 4301 Jones Bridge Rd, Bethesda, 20814 Maryland USA; 4 Flinders University, School of Medicine, GPO Box 2100, Adelaide, 5001 South Australia Australia; 5 Department of Medicine, Walter Reed National Military Medical Center, 8901 Wisconsin Ave, Bethesda, 20889 Maryland USA; 6 Department of Educational Development and Research, Maastricht University, Maastricht, 6200 MD The Netherlands

**Keywords:** Clinical reasoning, Medical education, Situated cognition, Quantitative methods

## Abstract

**Background:**

The impact of context on the complex process of clinical reasoning is not well understood. Using situated cognition as the theoretical framework and videos to provide the same contextual “stimulus” to all participants, we examined the relationship between specific contextual factors on diagnostic and therapeutic reasoning accuracy in board certified internists versus resident physicians.

**Methods:**

Each participant viewed three videotaped clinical encounters portraying common diagnoses in internal medicine. We explicitly modified the context to assess its impact on performance (patient and physician contextual factors). Patient contextual factors, including English as a second language and emotional volatility, were portrayed in the videos. Physician participant contextual factors were self-rated sleepiness and burnout.. The accuracy of diagnostic and therapeutic reasoning was compared with covariates using Fisher Exact, Mann-Whitney U tests and Spearman Rho’s correlations as appropriate.

**Results:**

Fifteen board certified internists and 10 resident physicians participated from 2013 to 2014. Accuracy of diagnostic and therapeutic reasoning did not differ between groups despite residents reporting significantly higher rates of sleepiness (mean rank 20.45 vs 8.03, U = 0.5, *p* < .001) and burnout (mean rank 20.50 vs 8.00, U = 0.0, *p* < .001). Accuracy of diagnosis and treatment were uncorrelated (*r* = 0.17, *p* = .65). In both groups, the proportion scoring correct responses for treatment was higher than the proportion scoring correct responses for diagnosis.

**Conclusions:**

This study underscores that specific contextual factors appear to impact clinical reasoning performance. Further, the processes of diagnostic and therapeutic reasoning, although related, may not be interchangeable. This raises important questions about the impact that contextual factors have on clinical reasoning and provides insight into how clinical reasoning processes in more authentic settings may be explained by situated cognition theory.

**Electronic supplementary material:**

The online version of this article (10.1186/s12909-017-1041-x) contains supplementary material, which is available to authorized users.

## Background

Clinical reasoning research has focused predominantly on diagnostic reasoning with less known about therapeutic reasoning. There are several potential reasons for this. First, the importance of improving diagnostic accuracy has been emphasized in a recent Institute of Medicine report [1]. Further, this report focuses on improving diagnosis to improve care with little specific attention to therapeutic reasoning or how diagnostic and therapeutic reasoning may contrast. Second, it is arguably easier to study a diagnostic label than a treatment plan, which must be molded to patient circumstances and preferences. Thus, therapeutic reasoning may be more value based and nuanced than assigning a diagnostic label. In addition, it has traditionally been thought that making an accurate diagnosis (or at least a short list of prioritized potential diagnoses) is an assumed intermediate step to arriving at a correct therapeutic plan. Taken together, these circumstances have translated into sparse knowledge on the factors that drive therapeutic reasoning.

Our understanding of clinical reasoning has emerged from multiple fields outside of medicine. Some of these frameworks, such as dual processing theory (e.g. non-analytic and analytic reasoning), emphasize the individual physician [2–4], which can minimize the potential role of the patient, the environment, and the interactions between the patient, the physician and the environment.

Context is defined as the “inter-related conditions in which something exists or occurs” [5]. This is more than a location but also how the individuals in a location interact with each other and the environment [6]. As such, context in a medical encounter can be divided into physician, patient and environmental factors [6]. Previous research by Brooks et al. has demonstrated that “contextual factors,” such as a diagnostic suggestion, can alter diagnostic accuracy [7]. Similarly, Eva and colleagues demonstrated that diagnostic suggestion can alter the consideration of other important alternative diagnoses [8]. In addition, we have demonstrated in prior investigations that patient contextual factors of low proficiency in English and challenging of a physician’s credentials, in addition to encounter contextual factors of incorrect diagnostic/therapeutic suggestion and atypical disease presentation, appear to be negatively associated with diagnostic reasoning in board-certified physicians when viewing a set of recorded clinical scenarios [9]. Further, studies have found that modifiable physician factors of sleep deprivation and burnout negatively relate to performance [10, 11].

**Fig. 1:**
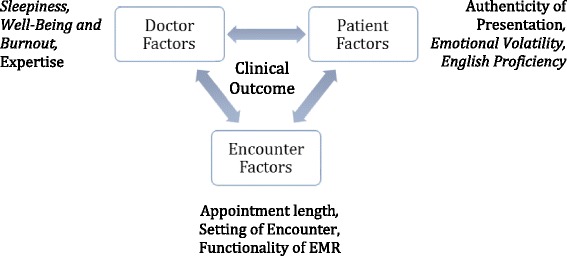
Situated cognition as a framework for context within a sample clinical encounter. The clinical outcome is dependent upon the complex interactions of all components; the physician, the patient, and the encounter. The types of factors evaluated in this study are shown in italics

Situated cognition provides a theoretical framework in which to understand the complex interplay of these contextual factors on diagnostic and therapeutic reasoning in more authentic settings than a classroom or on multiple-choice examinations, which have been the subject of few previous studies. Situated cognition is based upon the premise that reasoning and learning are situated within the physical environment and social context of an experience [12–14]. This theory contends that there are complex interactions shaped by the social context of participants and the environment in which the social interaction occurs. When considering clinical reasoning from a situated cognition perspective, one would assert that the outcome of a clinical encounter is not solely the product of the physician’s knowledge, but is instead an interplay of the evolving interactions between the physician, the patient and the environment in which the encounter occurs [13]. Other health professionals, such as a nurse or physical therapist, add additional interactions, which also have the potential to influence the outcome of the encounter. It also does not assume that diagnostic and therapeutic reasoning are the same. Situated cognition’s dynamic interactions that occur between the physician, patient and environment during a clinical encounter are shown in Fig. 1 [9].

We examined the relationship between specific patient and physician contextual factors on diagnostic and therapeutic reasoning accuracy in board certified internists and resident physicians (physicians in training). We postulated that the ability to arrive at a correct therapeutic plan would be contingent upon making a correct diagnosis and that, although related, diagnostic and therapeutic reasoning are not the same process. This is consistent with situated cognition in that a proper plan would be expected to be contingent upon the specifics of an encounter of a patient with a given diagnosis (the interactions between patient, physician, and environment) whereas more traditional approaches to clinical reasoning would suggest that diagnostic and therapeutic decisions should be highly correlated due to the choice of therapy being a linear product of the diagnosis [4]. We also hypothesized that because board certified internists have developed more elaborate illness scripts, their accuracy in both diagnostic and therapeutic reasoning would be superior to resident physicians even when presented with various contextual factors. Thus, we compared diagnostic and therapeutic reasoning accuracy between these two groups. We sought to address two gaps in the clinical reasoning literature: 1) the relationship (or lack thereof) between diagnostic and therapeutic reasoning and 2) the impact of selected patient and physician specific contextual factors on clinical reasoning performance.

Script theory asserts that during clinical encounters all physicians utilize prior gained knowledge to generate hypotheses and create actionable management plans [15]. The testing of illness scripts from the standpoint of specific contextual factors potentially seen in practice has not been the focus of prior work and would provide some evidence for situated cognition theory as well as build on illness script theory. As therapeutic reasoning is the step that most directly leads to patient help (or harm), better understanding this phenomenon and how it relates to diagnostic reasoning could assist educators and policy makers alike.

## Methods

### Participants

Residents, defined as physicians in an internal medicine residency-training program, and board certified internists were recruited for the study. All internal medicine resident physicians and board certified internists in the Uniformed Services University Capital Consortium in Bethesda, Maryland were invited to take part in the study. Subjects were contacted by email by a research assistant and invited to participate in the study. There were no exclusion criteria. No financial incentive was provided for participation in the study. The Institutional Review Board (IRB) of the Uniformed Services University approved this protocol and all participants signed an informed consent document prior to participation.

### Design

**Fig. 2:**
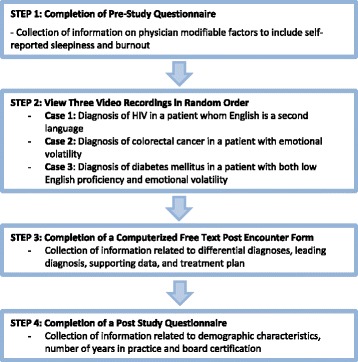
Flow chart demonstrating the overall structure of the study design

The study design has been used before and is part of a program of research with previously published methodology [9, 12]. Fig. 2 provides an overview of the steps applicable to participants that led to data collection. Participants viewed a series of video recorded standardized patient encounters 3 to 5 min in length in which both the patient and physician portrayed in the video were trained standardized actors and the same standardized actor served as the physician for all three videos. The cases were designed to represent straightforward disease presentations in the setting of varied contextual factors. In other words, the goal of the investigation was to determine if the manipulated contextual factors influence physician performance consistent with situated cognition theory. Case 1 portrayed a diagnosis of HIV in a patient for whom English is a second language. Case 2 portrayed a diagnosis of colorectal cancer in a patient presenting with emotional volatility. Case 3 portrayed a diagnosis of diabetes mellitus in a patient with both low English proficiency and emotional volatility. The videos were developed and subsequently vetted by a separate group of board certified physicians to help ensure authenticity in clinical practice and enable the explicit exploration of diagnostic and therapeutic reasoning.

Case construction involved discussions with experts in internal medicine from a variety of sub-specialties and experts in clinical reasoning to select diagnoses commonly seen in practice and describe straightforward presentations of these diagnoses. Next, common patient contextual factors were discussed. The cases were then written by a group of these experts and discussed with the larger team. Pilot testing was performed prior to insertion of contextual factors using the script for the subsequent video encounters to help ensure that the cases were common, relevant, and straightforward. Edits were made based on pilot testing and contextual factors were next incorporated into the scripts. Videos were believed to represent an optional means to study the impact of contextual factors on performance, as each participant would receive the same “stimulus.” Both verbal and nonverbal information could be conveyed in video scenarios and video recordings are of a level of authenticity needed to explore our research questions using situated cognition as a theoretical framework. These cases were piloted with a group of six internal medicine faculty prior to insertion of the contextual factors. This faculty group unanimously agreed that the cases represented straightforward disease processes seen in actual practice.

The impact of potentially modifiable physician factors was explored to include self-reported sleepiness and burnout. As illustrated in Fig. 2, participants first completed a pre-study questionnaire to assess self-reported sleepiness (Likert-type scale, 0 = never through 3 = high) and burnout (Likert-type scale, 0 = never through 4 = very often) utilizing previously validated instruments [16, 17]. The original scales from these instruments were used and were not modified for this study. Following this, participants viewed a series of three video recordings. These recordings portrayed one of three cases, each featuring a specific contextual factor as listed previously. In addition, each video portrayed applicable history and physical exam findings for each diagnosis. The order in which the videos were viewed was selected at random for each participant.

Following the viewing of each video recording, the participants completed a computerized free-text post-encounter form (PEF) (Additional file 1). The computerized PEF collected additional requested information to include differential diagnosis, leading diagnosis with supporting data, and treatment plan [18]. The use of this PEF for assessment of clinical reasoning has been previously established in medical students and the key for the PEF, as detailed above, was established through consensus discussion with a group of expert physicians [12, 19]. After completion of each PEF, participants completed a post-study questionnaire, which inquired about the participant’s demographic characteristics, number of years in practice, years of training and board certification.

### Data analysis

The primary outcome of analysis was accuracy of diagnostic and therapeutic reasoning as determined by performance on the PEF (Additional file 1). In terms of physician factors, we chose to use two recently cited and potentially modifiable covariates, self-reported sleepiness and self-reported burnout (Fig. 1) [16, 17]. We also explored performance based on components of the PEF form to include differential diagnosis and supporting data (Additional file 1). This was investigated at the level of each individual case and in aggregate. Each PEF was scored by two investigators using a previously established key (SJD, TR). An inter-rater reliability analysis using the Kappa statistic was performed. Disagreements in coding were resolved by consensus. Entries for each item of the PEF were assigned 0 points if incorrect, 1 point if partially correct and 2 points if correct based on pre-determined answers of correctness as agreed upon by the panel of experts who participated in the development of the case scripts. Missing data on the PEF, which indicated that a participant did not fill out an answer for questions related to items such as leading diagnosis, differential diagnoses, supporting data or treatment plan, were counted as incorrect. Descriptive statistics, including medians, were calculated for continuous variables and proportions were calculated for ordinal variables. To explore bivariate associations, Fisher Exact test was used for ordinal variables, and Mann-Whitney U test and Spearman correlation for continuous variables. All statistical analysis was done using SPSS 22.0.

## Results

A total of 15 board certified internists (12 males, 3 females) and 10 residents (5 males, 5 females) participated in this study from 2013 to 2014. For the group of board certified internists, the average year of medical school graduation was 1994 (SD ± 9) and the average year of board certification was 1997 (SD ± 9, range 1973–2008). All internists practiced in an academic setting. For residents, the average year of medical school graduation was 2009 (SD ± 3, range 2003–2012). The inter-rater reliability for coding of the PEF (SDR, TR) was Kappa = 0.93.

**Fig. 3:**
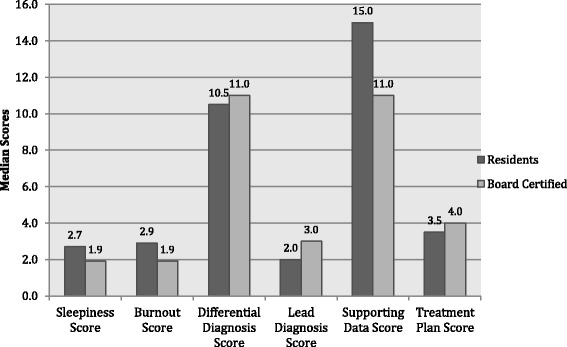
Median Values for Variables Used to Evaluate Differences in Diagnostic and Therapeutic Reasoning in Board Certified Versus Resident Physicians

A Mann-Whitney U test was performed to determine if there were differences in key covariates between resident and board certified physicians. The distributions of all covariates were assessed by visual inspection. The distributions were skewed and were not similar. Table 1 presents mean rank scores for covariates in the resident versus board certified participants. Median values for each covariate are presented in Fig. 3.

**Table 1:** Association of the Mean Rank for Variables Used to Evaluate Differences in Diagnostic and Therapeutic Reasoning in Board Certified Versus Resident Physicians

Covariate	Resident *n* = 10	Board Certified *n* = 15	U Statistic	*p*-value
Sleepiness Score	20.4	8.0	0.5	< 0.001
Burnout Score	20.5	8.0	0.0	< 0.001
Total Score Differential Diagnosis	13.8	12.5	67.0	0.57
Total Score Lead Diagnosis	11.1	14.3	56.0	0.37
Total Score Correct Supporting Data	14.7	11.9	58.0	0.20
Total Score Treatment Plan	13.1	13.0	74.0	0.89

As expected due to work hours while in training, the resident group had greater levels of self-reported sleepiness (mean rank 20.45 vs 8.03, U = 0.5, *p* < .001) and burnout (20.5 vs 8.0, U = 0.0, *p* < .001). There were no statistically significant differences between groups in total scores for treatment, leading diagnosis, total supporting data or differential diagnosis.

Table 2 presents correlations between total scores on leading diagnosis and treatment for both groups and covariates of interest to include self-reported sleepiness, self-reported burnout, and total scores for leading diagnosis, differential diagnosis and quality of total supporting data.

**Table 2:** Correlations Between Treatment Scores and Covariates Used to Evaluate Differences in Diagnostic and Therapeutic Reasoning in Board Certified Versus Resident Physicians

Spearman’s Rho Correlation	Resident Score Lead Diagnosis	Resident Total Score Treatment	Board Certified Score Lead Diagnosis	Board Certified Total Score Treatment
Sleepiness Score	0.52	0.57	0.14	−0.32
Burnout Score	0.57	0.50	0.37	0.10
Total Score Differential Diagnosis	−0.08	0.42	0.17	0.01
Total Score Lead Diagnosis	–	0.17	–	0.36
Total Score Correct Supporting Data	0.25	0.41	0.55*	0.86**

*p = 0.03

** *p* < 0.001

No significant correlations between total scores and these covariates were found in either group except that quality of supporting data in the board certified internists significantly correlated to scores on leading diagnosis (0.553, *p* = .03) and treatment plan (0.885, *p* < .001). Notably, no statistically significant correlation was found between total treatment score and leading diagnosis for either group. Correlations between the above listed covariates and the participants combined, as a single group, did not alter statistical significance.

Table 3 presents the proportion of internists and residents able to achieve a correct leading diagnosis or treatment plan for each video recorded clinical scenario.

**Table 3:** Correct Diagnosis and Treatment Plan by Case and in Aggregate as a Measure to Evaluate Differences in Accuracy of Diagnostic and Therapeutic Reasoning in Board Certified Versus Resident Physicians

Case	Resident (*n* = 10)	Board Certified (*n* = 15)	*p*-value
Case 1: HIV			
Correct Diagnosis	10% (1)	13% (2)	0.65
Correct Treatment Plan	30% (3)	27% (4)	0.60
Case 2: Colorectal Cancer			
Correct Diagnosis	40% (4)	53% (8)	0.40
Correct Treatment Plan	50% (5)	53% (8)	0.60
Case 3: Diabetes Mellitus			
Correct Diagnosis	40% (4)	53% (8)	0.40
Correct Treatment Plan	60% (6)	67% (10)	0.53
Correct Lead Diagnosis Across All Cases			0.50
3 cases correct	10% (1)	13% (2)	
2 cases correct	10% (1)	27% (4)	
1 case correct	40% (4)	27% (4)	
0 cases correct	40% (4)	33% (5)	
Correct Therapeutic Reasoning Across All Cases			0.87
3 cases correct	10% (1)	13% (2)	
2 cases correct	40% (4)	40% (6)	
1 case correct	30% (3)	27% (4)	
0 cases correct	20% (2)	20% (3)	

Overall, the proportion of participants able to achieve a correct response on the PEF for leading diagnosis was lowest in Case 1 (residents 10%, internists 13%) and highest in Case 3 (residents 40%, internists 53%) for both groups. The proportion of participants able to achieve a correct response for treatment plan was also lowest in Case 1 (residents 30%, internists 27%) and highest in Case 3 (residents 60%, internists 67%). In both groups, the proportion scoring a correct response for treatment plan was higher than the proportion scoring a correct response for diagnosis in most cases (Table 3). Analysis using Fisher Exact test demonstrated that the proportion achieving a correct response for leading diagnosis or treatment plan were not statistically significant between the two groups by case or in aggregate. Additionally, when evaluated on an individual participant level, there was variability in the ability to consistently achieve a correct diagnosis or treatment plan across cases (data not shown).

## Discussion

Our first hypothesis was that while diagnostic and therapeutic reasoning are related, they represent different tasks and thus correlations were expected to be small to moderate. This is based on situated cognition therapy. Traditionally, it has been thought that in order to develop a correct therapeutic plan, a physician must first establish a correct diagnosis. Notably, our results suggest that there was not a strong correlation between leading diagnosis and therapeutic plan. This suggests that although diagnostic and therapeutic reasoning may be related, they may not be the same process. As such, this raises the question as to whether a correct diagnosis is always needed in order to develop a correct therapeutic plan. In addition, there were low correlations between history, physical exam and differential diagnosis with diagnostic and therapeutic reasoning. We believe that exploring the lack of expected correlations represents an important area for potential future research. As Goldszmidt et al. [20] have recently proposed, diagnostic and therapeutic reasoning are not likely simple constructs but processes composed of multiple tasks. Their proposed list of 24 clinical reasoning tasks thought to occur during a clinic encounter implies an inter-related relationship between potentially separate processes of diagnostic and therapeutic reasoning.

Our second hypothesis that board certified physicians would be superior to resident physicians in both diagnostic and therapeutic accuracy was not supported. Thus, using the lens of situated cognition, it appears that the presence of contextual factors may have altered the accuracy of diagnostic and therapeutic reasoning in both groups given the straightforward nature of the cases. The contextual factors displayed in the videos were previously shown to impact the performance of board certified physicians [9]. One possible explanation regarding the failure to find board certified physicians superior in diagnostic or therapeutic accuracy is that resident physicians may be less susceptible to the impact of contextual factors and this counterbalanced the effect of relatively less well-developed illness scripts. It may be that generational differences in medical education between resident and board certified physicians, such as earlier introduction to patient care, use of simulation based learning, or new approaches to the development of cultural competency, have influenced the context and frequency of interpersonal experiences for residents. The exposure to these experiences may impart resiliency against the influence of contextual factors on clinical reasoning. This would imply that both rich content knowledge and experience in dealing with contextual factors play important roles in clinical reasoning; scripts are modified through practice and the presence of contextual factors may impact this process of modification which would be consistent with situated cognition theory. It is recognized that the failure to detect differences between the groups could also be the result of the relatively small sample size in this investigation and should be confirmed in larger studies.

In board certified physicians there was positive correlation between correct supporting data and accuracy of diagnosis and treatment plan that was not present in resident physicians (Table 2). This may be the result of board certified physicians having more sophisticated illness scripts [15]. Script theory contends that all physicians utilize prior gained, relevant knowledge during clinical encounters. This entails recognition of important details, which help generate hypotheses and creation of actionable plans [15]. Situated cognition theory would argue that there is no set order in which this occurs. Taken further, this framework promotes the idea that reasoning is dynamic and can change based upon the interactions present and how information is revealed or acquired within each specific context. This explanation could account for variability in data collection amongst physicians, who proceed through a clinical encounter in a way that is most efficient according to activation of the individual’s own illness scripts [15].

This study was limited in a number of ways. First, while consistent with many studies in the area of clinical reasoning, the sample size was small. Detecting a moderate effect size the post hoc power analysis demonstrated β = 0.24. Although the power is small, the findings of vulnerability in clinical reasoning due to the influence of contextual factors and the unexpected differences in diagnostic and therapeutic reasoning warrant further study. Due to the time intensive nature of our design, multi-institutional involvement for additional subject recruitment is likely needed to optimize power.

Second, our findings represent correlation and not causation. While evidence for validity of our PEF was gathered in students and was vetted by expert clinicians coming to consensus, the PEF used represents one clinical reasoning outcome. Third, while previous evidence supports the impact of the contextual factors used in this study in board certified physicians [9], we did not include a control condition to explicitly replicate this finding. Fourth, while watching a video and filling out a post encounter form is not identical to practice, we believe that the use of videos portraying contextual factors in an identical fashion (same “stimulus” for all participants) provides a more authentic way to assess the impact of contextual factors on practice. Fifth, low correlations between accuracy of diagnosis and treatment may, in part, be due to the limited reliability of our outcome measures, to include the use of self-assessment measures. Finally, the clinical cases utilized assessed clinical reasoning as it pertains to three diagnoses, which represents only a small sample of disease processes.

## Conclusions

This study raises important questions about the impact that contextual factors have on diagnostic and therapeutic reasoning. This study also underscores that the processes of diagnostic and therapeutic reasoning, although related, may not be interchangeable and that a correct diagnostic assessment may not always be necessary in order to arrive at a correct therapeutic plan. Further research is needed to determine what comprises diagnostic and therapeutic reasoning in clinical practice, how these processes occur, and how these can best be taught and assessed. Exploring the expected correlations, or lack thereof, of diagnostic and therapeutic reasoning represents an important area for potential future research.

## Additional files

Additional file 1: Post Encounter Form. (PDF 45 kb)

## Abbreviations

HIV: Human immunodeficiency virus
IRB: Institutional review board
PEF: Post encounter form
SD: Standard deviation
