# Supplementary material for: Contextual factors and clinical reasoning: differences in diagnostic and therapeutic reasoning in board certified versus resident physicians
Source: BMC Med Educ. 2017 Nov 15;17:211. doi: 10.1186/s12909-017-1041-x (PMC5688653; doi:10.1186/s12909-017-1041-x)
Supplement: Supplementary file 1 — Post Encounter Form. (PDF 45 kb) [file 12909_2017_1041_MOESM1_ESM.pdf]

Additional File 1: Post Encounter Form

1. What else do you want to ***ask*** this patient? *(List one to five questions)*

2. What else would you want to ***look for*** on this patient's physical exam? *(List one to five items)*

3. Write a complete problem list.

4. What is your ***differential diagnosis***? (Please list in order of likelihood and list at least 3 responses)

   a.
   b.
   c.
   d.
   e.
   f.

5. What is your ***leading diagnosis*** and what data supports this diagnosis? *(List one to five pieces of evidence).*

Diagnosis	Data that supports this diagnosis

6. What is your ***treatment plan*** for this patient? (Diagnostic and/or therapeutic).

Work up	Therapy
